# A phase-II study based on dose adjustment according to UGT1A1 polymorphism: is irinotecan underdosed in first-line FOLFIRI regimen for mCRC?

**DOI:** 10.1007/s00280-023-04603-x

**Published:** 2023-11-07

**Authors:** Angeline Ginzac, Emilie Thivat, Caroline Petorin, Damien Richard, Pauline Herviou, Ioana Molnar, Hervé Devaud, Isabelle Creveaux, Florent Ferrer, Nicolas Authier, Marine Jary, Denis Pezet, Xavier Durando

**Affiliations:** 1 INSERM U1240 Imagerie Moléculaire et Stratégies Théranostiques (IMoST), Université Clermont Auvergne, 63000 Clermont-Ferrand, France; 2 Centre d’Investigation Clinique, UMR501, 63000 Clermont-Ferrand, France; 3 Département de Recherche Clinique, Délégation Recherche Clinique et Innovation, Centre Jean PERRIN, 63000 Clermont-Ferrand, France; 4 Département de Chirurgie Digestive et Hépatobiliaire, Hôpital Estaing, 63000 Clermont-Ferrand, France; 5 Service de Pharmacologie Médicale, Unité de Pharmacologie et de Toxicologie Biologique, CHU Gabriel MONTPIED, 63000 Clermont-Ferrand, France; 6 Service d’oncologie Médicale, Centre Jean PERRIN, 63000 Clermont-Ferrand, France; 7 Département de Biochimie et Génétique Moléculaire, CHU Clermont-Ferrand, 63000 Clermont-Ferrand, France; 8 Université Clermont Auvergne, CHU de Clermont-Ferrand, Inserm, Pharmacologie Médicale/Centre Evaluation et Traitement de La Douleur, Observatoire Français des Médicaments Antalgiques, Institut Analgesia, 63001 Clermont-Ferrand, France; 9 Service de Chirurgie Digestive, U1071, M2iSH, USC-INRA 2018, CHU Clermont-Ferrand, Université Clermont Auvergne, INSERM, INRA, F-63000 Clermont-Ferrand, France

**Keywords:** Irinotecan, UGT1A1 polymorphism, Metastatic colorectal cancer, First-line treatment

## Abstract

**Purpose:**

Irinotecan has considerable importance in the treatment of metastatic colorectal cancer (mCRC). UDP-glucoronyltransferase (UGT) 1A1 is responsible for the inactivation of SN-38, a metabolite of irinotecan. Depending on UGT1A1 polymorphism, the activity of the UGT enzyme can be reduced leading to more frequent occurrence of adverse events related to irinotecan. The present study aimed to assess the safety and efficacy of different doses of irinotecan adjusted according to UGT1A1 polymorphism.

**Methods:**

Thirty-four patients treated with FOLFIRI as first-line treatment for mCRC were included in this study. The irinotecan dosage was adapted on the basis of UGT1A1 polymorphisms: *1/*1 (370 mg/m^2^); *1/*28 (310 mg/m^2^), and *28/*28 (180 mg/m^2^). The incidence of grades 3 and 4 toxicities (neutropenia, febrile neutropenia, and diarrhoea) was recorded. Response was assessed according to the RECIST 1.1 criteria.

**Results:**

On the basis of UGT1A1 genotyping, 20 patients were *1/*1 (58.8%), 12 were *1/*28 (35.3%) and 2 were *28/*28 (5.9%). Seven patients experienced at least one severe toxicity, i.e., 21% of the population, amounting to eleven adverse events. Concerning the response rate, 15 patients (44%) had partial or complete response.

**Conclusion:**

This study demonstrates that mCRC patients treated with FOLFIRI can tolerate a higher dose of irinotecan than the standard dose, i.e., > 180 mg/m^2^, on the basis of their UGT1A1 genotype, without increased toxicities.

**Trial registration:**

NCT01963182 (registered on 16/10/2013, Clermont-Ferrand, France).

## Introduction

Colorectal cancer is the third most commonly diagnosed cancer worldwide and the second leading cause of cancer-related death [1]. Approximately 25% of colorectal cancer patients are at the metastatic stage at the time of diagnosis and metastatic disease occurs in 40 to 60% of new cases. Irinotecan has considerable importance in the treatment of metastatic colorectal cancer (mCRC). It is generally used in combination with 5-fluorouracil and targeted therapies (cetuximab, bevacizumab or panitumumab), at various doses. The FOLFIRI regimen (400 mg/m^2^ bolus IV on day one and 2400 mg/m^2^ IV on day two of 5-fluorouracil, 200 mg/m^2^ leucovorin, and 180 mg/m^2^ irinotecan) is currently used as first-line treatment for mCRC with an objective response rate of 40% and median progression-free survival (PFS) of 7 months [2]. Dose-limiting toxicities (DLT) with irinotecan are neutropenia and diarrhoea, predominantly caused by the active metabolite of irinotecan, SN38. The level of SN38 is regulated by glucuronidation in inactive SN38 glucuronide (SN38-G) via uridine diphosphate glucuronosyltransferase (UGT1A1). Different levels of SN38 glucuronidation could explain the inter-individual variation in the pharmacokinetic parameters of SN38, and the various toxicities observed after the administration of irinotecan. Indeed, genetic polymorphism in the gene promoter (UGT1A1*28) causes a sub-expression of the enzyme, associated with circulating concentrations of SN38 above those observed for UGT1A1*1 patients [3]. The frequency of allele 28 is commonly found in African and European population but is rare in Asian population (respectively about 0.426, 0.387 and 0.160) [4, 5]. In European population, this mutation is present in 7.7 to 8.8% of cases for genotype *28/*28, in 41.9 to 45.6% for genotype *1/*28, and in 45.6% to 50.5% for genotype *1/*1 [6].

Several studies have evaluated the efficacy and safety of different irinotecan doses based on the UGT1A1 genotype [2, 3, 7–9]. The results suggest that patients with at least one wild-type allele (*1/*1 or *1/*28) can tolerate higher doses, i.e., > 180 mg/m^2^. Dose optimization based on UGT1A1 polymorphism could enable the individualization of treatment. As irinotecan is a dose-dependent chemotherapy, dose adjustment could enable at least a similar response rate with limited toxicity. Our study aimed to evaluate the feasibility of irinotecan dose adjustment according to UGT1A1 polymorphisms in a population of mCRC patients. In addition, pharmacokinetic analyses were expected to provide a better understanding of the mechanisms involved in response-to-treatment variations and the occurrence of adverse events.

## Methods

### Patients

Eligible patients were male or female (> 18 years old) with histologically or cytologycally proven colorectal cancer, an indication of treatment with FOLFIRI ± bevacizumab or cetuximab or panitumumab, the presence of at least one measurable target according to the RECIST criteria, life expectancy of at least 3 months and adequate biological functions (renal, hepatic and haematological). Patients already treated for metastatic disease, taking anti-epileptics drugs, allergic or intolerant to irinotecan, having a contraindication for 5-FU, and with chronic inflammatory bowel disease or bowel obstruction, were excluded. All patients provided written informed consent prior to enrolment.

### Study design

The study was approved by the local ethics committee and the competent authority at national level (Agence Nationale de Sécurité des Médicaments et des produits de santé) and registered online at ClinicalTrial.gov (https://clinicaltrials.gov/ct2/show/NCT01963182, 16/10/2013).

This was a prospective phase-II, multicentre, non-randomized trial aiming to demonstrate the feasibility of an individualized dose of irinotecan on the basis of genetic polymorphisms in first-line treatment for mCRC involving the FOLFIRI regimen.

### Genotyping

The UGT1A1 genotype was determined within 7 days before the first cycle of treatment, so as to adapt the dose of irinotecan. This analysis was performed by PCR (FRET) with a LightCycler® 2.0 FastStart DNA Master HybProbe (Roche Diagnostics, Meylan, France) and UGT1A1 Light SniP (TIB MOLBIOL, Berlin, Germany). DNA was extracted using QIAamp® DNA Mini and Blood 50 (QIAGEN, Germany).

### Treatment

Patients were treated twice a month with the FOLFIRI regimen until progression. The FOLFIRI treatment comprised: 5FU IV bolus 400 mg/m^2^ on day 1, 5FU infusion 2400 mg/m^2^ on days 1 and 2, folinic acid 400 mg/m^2^ on day 1 and irinotecan at the UGT1A1 genotype-adapted dosage on day 1 (180 mg/m^2^ for UGT1A1 *28/*28, 310 mg/m^2^ for *1/*28, and 370 mg/m^2^ for *1/*1 genotype, on the basis of the results of the phase-I study conducted by Toffoli et al. [3]). Patients could receive 5 mg/kg of bevacizumab, 500 mg/m^2^ of cetuximab or 6 mg/kg of panitumumab, before the FOLFIRI regimen every 2 weeks. Prophylactic G-CSF administration was not allowed as primary prevention.

#### Dose reduction and discontinuation rules

In case of grade 4 neutropenia, thrombocytopenia or febrile neutropenia, treatment was delayed for 1-–2 week(s) to allow a return to normal (neutrophil count > 1500/mm^3^ and platelet count > 100,000/mm3) before readministering irinotecan. If the required counts were not achieved on the day of the theoretical recovery (J7 and J15), the irinotecan dose was reduced by 20%. The same process was repeated, with 20% of reduction in dose if haematological recovery was not achieved at the theoretical moment in the cycles. In case of non-haematological grades 3–4 toxicities associated with irinotecan in the inter-treatment period, we waited until the symptoms disappeared completely before administering the medication and proceeded to a dose reduction of 20% in the next cycle. The reduced doses were maintained for all subsequent cycles.

#### Concomitant care

Granulopoiesis-stimulating factors were administered as a secondary prevention for FOLFIRI-treated patients with grade 4 neutropenia or febrile neutropenia. All symptomatic treatments required for the comfort of the patients were allowed. Concomitant treatments prescribed during the study were at the investigator’s discretion and were to be reported. Any other anti-cancer treatments were prohibited, including any cytotoxic treatment and any hormonal therapy, apart from oestrogen-progestin contraception.

### Study objectives and endpoints

The primary objective was to evaluate the benefit in terms of toxicities and response to the individualization of the irinotecan dose according to UGT1A1 polymorphism. The secondary objectives were the pharmacokinetic study of irinotecan, SN38, SN38-G, and bevacizumab, and the evaluation of treatment efficacy.

The primary endpoint was assessed on the basis of the frequency of severe toxicities (defined as grade 4 neutropenia, grades 3 and 4 febrile neutropenia or grade 4 diarrhoea according to the NCI-CTC criteria v.4) and response rate (via imagery) according to the RECIST criteria v.1.1.

For the secondary outcomes, we investigated blood concentrations and the pharmacokinetic AUC of irinotecan, SN38, SN38-G, and bevacizumab, PFS, and response duration.

PFS was defined as the time between diagnosis and progression or death whichever occurred first. Overall survival (OS) was defined as the time between diagnosis and death. Patients were censored at the time of their last recorded follow-up if they were still alive.

### Pharmacokinetics

During the first cycle, the pharmacokinetics of irinotecan SN38, SN38-G, and bevacizumab (if present) were explored. Blood samples to monitor treatment exposure (AUC) were regularly collected at several time points:

- T0, T30, T60, T90, T120, T240, T480, T720, and T1440 min for FOLFIRI alone and FOLFIRI + vectibix
- T0, T30, T60, T90, T120, T180, T210, T240, T270, T300, T420, T660, T900, and T1620 for FOLFIRI + Erbitux
- T0, T30, T60, T120, T150, T180, T210, T330, T570, T810, and T1530 for FOLFIRI + Avastin

The plasma concentrations of these molecules were determined using high-performance liquid chromatography coupled with tandem mass spectrometer (HPLC–MS/MS), composed of a TSQ Quantum Ultra™ mass spectrometer (Thermo Fisher Scientific) coupled with Transcend TLX-1 liquid chromatography (Thermo Fisher Scientific). Both molecules were assayed after simple precipitation and the bio-analytical method was validated following ISO-1589 and EMEA guidelines [10]. The Limits of Quantification (LOQ) were 25 ng/ml and 5 ng/mL for irinotecan and SN38, respectively. These analyses were centralized and performed in the clinical pharmacology and toxicology laboratory of Clermont-Ferrand University Hospital.

Irinotecan, SN38, and SN38-G AUCs were calculated using the trapezoidal rule and then compared according to the patients’ UGT1A1 genotype status (*1/*1, *1/*28 and *28/*28) and the dose administered (370 mg/m^2^, 310 mg/m^2^ or 180 mg/m^2^).

### Statistical analysis

Given the trade-off between treatment efficacy and treatment toxicity, a two-stage Bryant and Day design was used to plan the study [11]. Concerning the response rate, for the lower limit for rejection we chose a response rate under 35%, and for the upper limit for acceptation a response rate of over 60%. For toxicity, for the lower limit for rejection we chose a toxicity rate over 40% and for the upper limit for acceptation a toxicity rate of under 20%.

A first positive interim analysis involving 16 patients enabled the study to be continued until 47 patients were included. The sample size for the final analysis was *n* = 34, as some patients were not assessable. Toxicity and efficacy assessments were performed using one-sided exact binomial tests. Toxicity frequency was computed using the maximum grade method.

All descriptive analyses were performed on the whole assessable population (*n* = 34), and in each UGT1A1 genotype-defined subgroup. AUC distributions of irinotecan, SN38, and SN38-G were compared between groups using Wilcoxon–Mann–Whitney tests. Survival data were analysed using the Kaplan–Meier method. Significance level was set at 5%. Statistical analyses were performed using R software (version 4.1.0; R Core Team, 2021).

## Results

### Patient characteristics

**Fig. 1:**
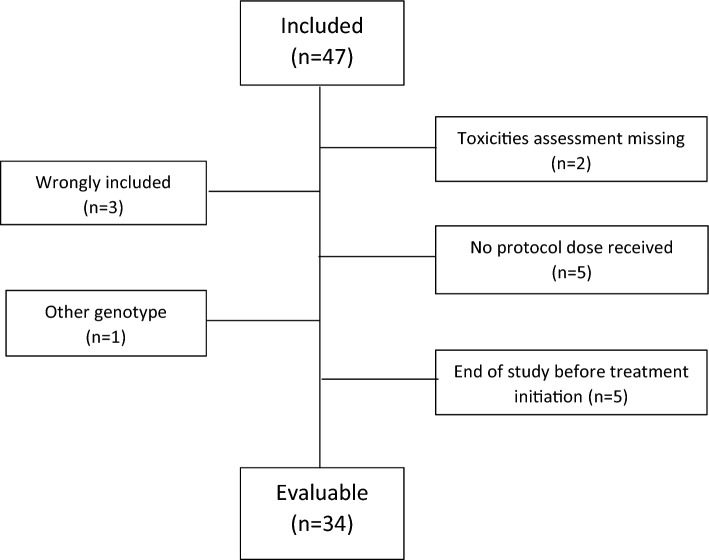
Patient flowchart

From April 2014 to November 2018, 47 patients were included in the study. Thirty-four were assessable for the analysis (Fig. 1). The patient characteristics are presented in Table 1. The median age was 67 years. There were 18 women (52.9%) and 16 men (47.1%). The main primary cancer was colon cancer (61.8%). Among the previous treatments received for the cancer, 47.1% (*n* = 16) had undergone surgery, 20.6% (*n* = 7) had received a course of chemotherapy, and 8.8% (*n* = 3) had received radiotherapy. On the basis of UGT1A1 polymorphisms, patients were classified into three groups: 58.8% (*n* = 20) were wild-type (*1/*1), 35.3% (*n* = 12) were heterozygous (*1/*28), and 5.9% (*n* = 2) were homozygous (*28/*28).

**Table 1:** Patient characteristics at baseline

	Whole sample (*n* = 34)	*1/*1 (*n* = 20)	*1/*28 (*n* = 12)	*28/*28 (*n* = 2)
General characteristics				
Age (years), median [range]	67.0 [34; 81]	65.5 [34; 81]	71.0 [42; 81]	73 [66; 80]
Gender, *n* (%)				
Women	18 (52.9)	10 (50)	8 (66.7)	0 (0)
Men	16 (47.1)	10 (50)	4 (33.3)	2 (100)
Body surface (m^2^), mean [range]	1.75 [1.47; 2.04]	1.74 [1.47; 2.04]	1.72 [1.47; 1.92]	2.02 [2.00; 2.04]
Disease characteristics				
Primary cancer, *n* (%)				
Colon	21 (61.8)	14 (70)	5 (41.7)	2 (100)
Rectum	7 (20.6)	3 (15)	4 (33.3)	0 (0)
Rectum and sigmoid	1 (2.9)	1 (5)	0 (0)	0 (0)
Sigmoid	5 (14.7)	2 (10)	3 (25)	0 (0)
KRAS mutation, *n* (%)				
Yes	17 (51.5)	8 (42.1)	8 (66.7)	1 (50)
No	16 (48.5)	11 (57.9)	4 (33.3)	1 (50)
No. of metastatic sites				
1	11	7	4	0
≥ 2	23	13	8	2
Metastatic sites, *n* (%)				
Node	11 (32.4)	8 (40)	2 (16.7)	1 (50)
Liver	27 (79.4)	15 (75)	10 (83.3)	2 (100%)
Bone	2 (5.9)	1 (5)	1 (8.3)	0 (0)
Lung	14 (41.2)	10 (50)	3 (25)	1 (50)
Peritoneum	3 (8.8)	2 (10)	1 (8.3)	0 (0)
Adrenal glands	4 (11.8)	2 (10)	2 (16.7)	0 (0)
mCRC types				
Metachronous metastasis	9 (26.5)	5 (25)	4 (33.3)	0 (0)
Synchronous metastasis	25 (73.5)	15 (75)	8 (66.7)	2 (100)
Previous treatment				
Surgery, *n* (%)				
Yes	16 (47.1)	8 (40)	7 (58.3)	1 (50)
No	18 (52.9)	12 (60)	5 (41.7)	1 (50)
Chemotherapy, *n* (%)				
Yes	7(20.6)	4 (20)	3 (25)	0 (0)
No	27 (79.4)	16 (80)	9 (75)	2 (100)
Radiotherapy, *n* (%)				
Yes	3 (8.8)	1 (5)	2 (16.7)	0 (0)
No	31 (91.2)	19 (95)	10 (83.3)	2 (100)

### Treatment

Nineteen patients (55.9%) received target agent combined with the FOLFIRI regimen (bevacizumab, cetuximab, and panitumumab or cetuximab + panitumumab): 12 in the *1/*1 group, 6 in the *1/*28 group, and 1 in the *28/*28 group.

Twenty-one patients (61.8%) had at least one cycle delayed: 11 (55%) in the *1/*1 group, 8 (66.7%) in the *1/*28 group, and 2 (100%) in the *28/*28 group. The median number of delayed cycles for patients receiving a higher dose of irinotecan than the standard dose, i.e., for the *1/*1 and *1/*28 subgroups was 2, while for those receiving the standard dose, i.e., *28/*28, it was 4. The main reasons were haematological and non-haematological toxicities (61.9%) followed by intercurrent events not related to the study (28.6%). Likewise, 20 patients (58.8%) had at least one irinotecan dose reduction: 10 (50%) in the *1/*1 group, 9 (75%) in the *1/*28 group, and 1 (50%) in the *28/*28 group. The reasons for dose reduction were non-haematological toxicity (61.1%), haematological toxicity (27.8%), and intercurrent events not related to the study (11.1%) (Table 2).

**Table 2:** Treatment information

	Total (*n* = 34)	1*/1* (*n* = 20)	1*/28* (*n* = 12)	28*/28* (*n* = 2)
Cycles administered at protocol dose				
Mean	12.65	10.40	11.83	40
Median [min–max]	10.5 [2–68]	6 [2–39]	12 [3–23]	40 [12–68]
Irinotecan dose (mg/m^2^)				
Mean	302	332	278	145
Median [min–max]	310 [34–444]	344 [34–444]	275 [236–310]	145 [109–180]
Cumulative dose of irinotecan (mg/m^2^)				
Mean	3544	3645	3167	4796
Median [min–max]	2660 [370–14430]	2220 [370–14430]	3288 [810–5852]	4796 [2160–7432]
Delayed cycles				
Number of cycles delayed	2	2	2	4
Number of patients concerned	21 (61.8%)	11 (55%)	8 (66.7%)	2 (100%)
Reasons				
Haematological toxicity	9 (42.9%)	7 (63.6%)	2 (25%)	0 (0%)
Non-haematological toxicity	2 (9.5%)	1 (9.1%)	1 (12.5%)	0 (0%)
Both haematological and non-haematological toxicity	4 (19%)	1 (9.1%)	2 (25%)	1 (50%)
Not related to the study	6 (28.6%)	2 (18.2%)	3 (37.5%)	1 (50%)
Irinotecan dose reduction				
Number of cycles with dose reduction	2	2	1	3
Number of patients concerned	20 (58.8%)	10 (50%)	9 (745%)	1 (50%)
Reasons				
Haematological toxicity	5 (27.8%)	1 (12.5%)	3 (33%)	1 (100%)
Non-haematological toxicity	11 (61.1%)	6 (75%)	5 (55.6%)	0 (0%)
Not related to the study	2 (11.1%)	1 (12.5%)	1 (11%)	0 (0%)

The median number of cycles received in the overall population was 11.50 (range [3; 68]). According to subgroups, there were 6 cycles [3; 39], 12 [3; 23], and 40 cycles [12; 68] for *1/*1, *1/*28 and *28/*28, respectively. Median relative dose intensity (RDI) was 87% (IQI 77.5–94.1): 89% (IQI 83–94) in group *1/*1, 80% (IQI 76–95) in group *1/*28, and 75% (min–max 57–93) in group *28/*28. Nineteen patients (56%) had an RDI of at least 85% (65% in group *1/*1, 42% in group *1/*28, and 50% in group *28/*28).

### Safety outcomes

Twenty-one per cent (*n* = 7) of the overall population experienced at least one severe adverse event: 3 patients in the *1/*1 cohort (15%), 3 patients in the *1/*28 cohort (25%), and 1 patient in the *28/*28 cohort (50%), which led us to conclude that severe toxicity concerned under 40% (exact binomial test, *p* = 0.014). Among the subgroups receiving a higher dose of irinotecan (*1/*1 and *1/*28), the proportion of severe adverse events was 19% (*n* = 6) (less than the 40% threshold, exact binomial test, *p* = 0.009). Seven cases of grade 4 neutropenia, two cases of grade 4 diarrhoea, two and one cases of grades 3 and 4 febrile neutropenia, respectively were observed. Table 3 presented the summary of number of events according cohort. A synthesis of all the hematologic and non-hematologic toxicities that occurred during the study is reported in Table 4.

**Table 3:** Summary of severe toxicities observed among cohorts

Toxicity	Grade	Cohort		
		*1/*1 (*n* = 3)	*1*28 (*n* = 3)	*2/*28 (*n* = 1)
Neutropenia	4	4	2	1
Febrile neutropenia	3	1	1	0
	4	0	1	0
Diarrhea	4	0	0	1

**Table 4:** Adverse events

Adverse event	Grade	Whole sample (*n* = 34)		*1/*1 (*n* = 20)		*1/*28 (*n* = 12)		*28/*28 (*n* = 2)	
		*n*	%	*n*	%	*n*	%	*n*	%
Non-hematologic events									
Nausea/vomiting	1–2	18	52.9	8	40	9	75	1	50
	3–4	10	29.4	7	35	3	25	0	0
Gastro-intestinal event	1–2	25	73.5	16	80	8	66.7	1	50
	3–4	5	14.7	2	10	2	16.7	1	50
Ear-nose-throat event	1–2	19	55.9	13	65	5	41.7	1	50
	3–4	4	11.8	2	10	1	8.3	1	50
Anorexia	1–2	20	58.8	10	50	9	75	1	50
	3–4	4	11.8	4	20	0	0	0	0
Asthenia	1–2	28	82.4	15	75	11	91.7	2	100
	3–4	5	14.7	4	20	1	8.3	0	0
Dermatological event	1–2	16	47.1	9	45	5	41.7	2	100
	3–4	1	2.9	0	0	1	8.3	0	0
Pain	1–2	9	26.5	5	25	3	25	1	50
Dyspnea	1–2	1	2.9	1	5	0	0	0	0
	3–4	1	2.9	1	5	0	0	0	0
Alopecia	1–2	4	11.8	2	10	2	16.7	0	0
Infection	1–2	9	26.5	3	15	6	50	0	0
	3–4	4	11.8	1	5	2	16.7	1	50
Neurological event	1–2	5	14.7	3	15	1	8.3	1	50
Hematologic events									
Thrombocytopenia	1–2	8	23.5	5	25	2	16.7	1	50
Anemia	1–2	24	70.6	13	65	9	75	2	100
	3–4	1	2.9	1	5	0	0	0	0
Hypokalemia	1–2	4	11.8	1	5	3	25	0	0
	3–4	1	2.9	1	5	0	0	0	0
Leukopenia	1–2	25	73.5	15	75	8	66.7	2	100
	3–4	2	5.9	0	0	2	16.7	0	0
Lymphopenia	1–2	4	11.8	2	10	2	16.7	0	0
	3–4	4	11.8	2	10	2	16.7	0	0
Neutropenia	1–2	8	23.5	5	25	3	25	0	0
	3–4	15	44.1	10	50	4	33.3	1	50
Febrile neutropenia	3–4	3	8.8	1	5	2	16.7	0	0

### Pharmacokinetics

Only patients with the dose corresponding to their UGT1A1 genotype status (i.e., 370 mg/m^2^ for *1/*1, 310 mg/m^2^ for *1/*28 and 180 mg/m^2^ for *28/*28) were retained for AUC comparisons in relation to UGT1A1 polymorphism. Therefore, out of the 34 patients included in this study, the AUC was evaluated for only 30 patients.

**Fig. 2:**
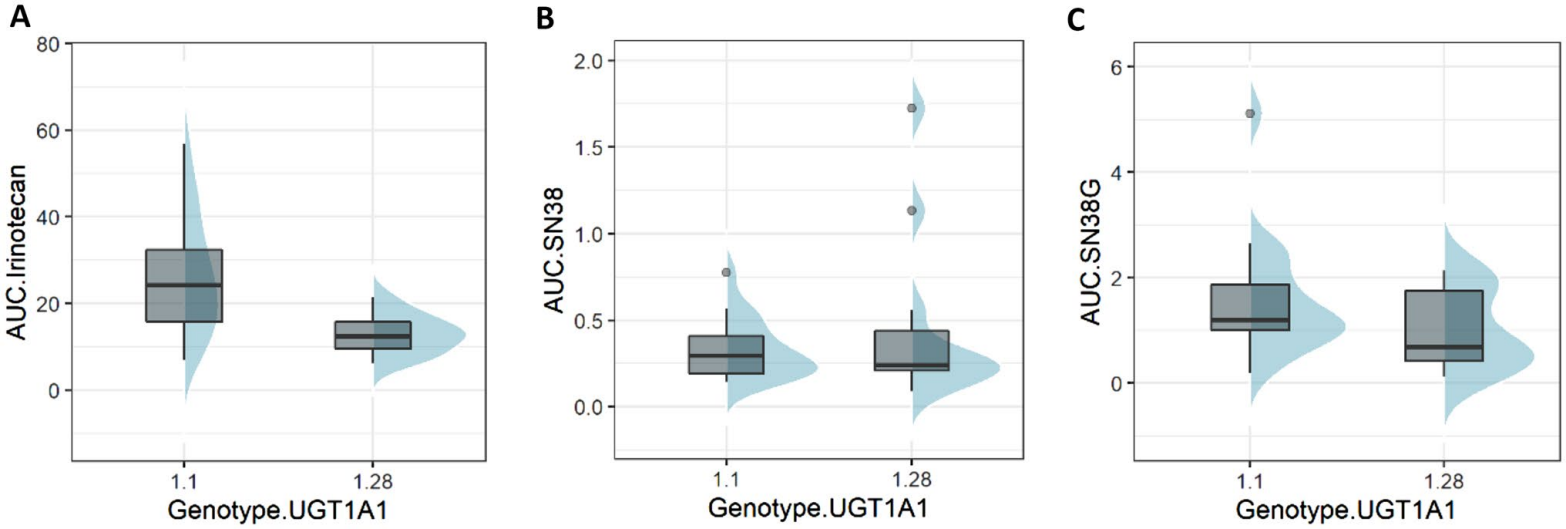
Variability in AUC values for irinotecan, SN38 and SN38-G. **A** Boxplot: AUC irinotecan; **B** boxplot: AUC SN38; **C** boxplot: AUC SN38-G

**Fig. 3:**
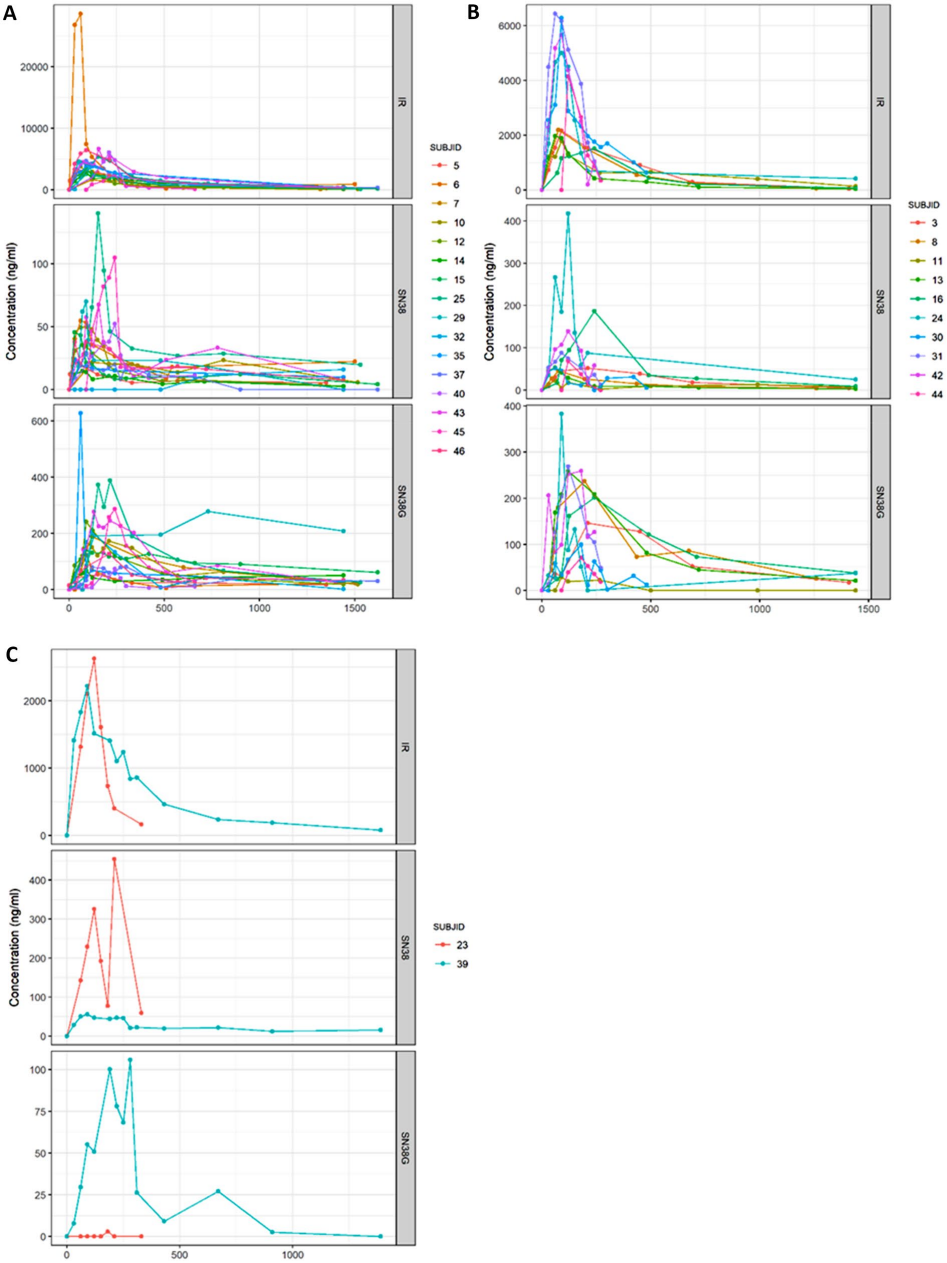
Pharmacokinetic curves for irinotecan, SN38 and SN38-G. **A** Cohort 1.1; **B** cohort 1.28; **C** cohort 28.28

Mean calculated AUCs for irinotecan, SN38, and SN38-G in relation to UGT1A1 polymorphism (*1/*1, *1/*28, and *28/*28) are summarized in Table 5. Figures 2A–C and 3A–C show the variability in AUC values for irinotecan, SN38, and SN38-G recorded among *1/*1, *1/*28 and *28/*28 patients. Boxplots for the *28/*28 patients were not calculated because of the small number of patients (*n* = 2).

**Table 5:** Pharmacokinetics

Variable	Cohort	*n*	Mean	Med	SD	Min	Max	IQI
AUC.irinotecan (µg h/mL)	Total	30	20.49	16.34	13.06	6.25	56.88	(11.77–24.97)
	1.1	17	26.77	24.16	14.02	6.91	56.88	(15.78–32.33)
	1.28	11	12.88	12.41	4.59	6.25	21.43	(9.53–15.78)
	28.28	2	9.01	9.01	3.66	6.42	11.60	(7.72–10.3)
AUC.SN38 (µg h/mL)	Total	30	0.43	0.29	0.41	0.09	1.72	(0.2–0.5)
	1.1	17	0.33	0.29	0.18	0.14	0.78	(0.19–0.41)
	1.28	11	0.47	0.24	0.51	0.09	1.72	(0.21–0.44)
	28.28	2	1.10	1.10	0.84	0.51	1.70	(0.8–1.4)
AUC.SN38G (µg.h/mL)	Total	30	1.39	1.11	1.06	0.13	5.12	(0.62–1.94)
	1.1	17	1.58	1.19	1.14	0.19	5.12	(1–1.86)
	1.28	11	1.01	0.69	0.75	0.13	2.14	(0.41–1.75)
	28.28	2	1.83	1.83	1.91	0.48	3.18	(1.16–2.51)
Glucuronidation.ratio	Total	28	4.25	3.51	3.05	0.25	12.98	(1.88–6.52)
	1.1	16	5.22	4.18	3.10	1.00	12.98	(3.37–6.78)
	1.28	10	3.25	2.38	2.71	0.25	9.09	(1.62–3.69)
	28.28	2	1.42	1.42	0.64	0.96	1.87	(1.19–1.64)
Biliary.index	Total	27	7.03	5.32	7.11	0.84	36.74	(2.91–9.65)
	1.1	15	6.20	5.14	4.39	1.30	14.22	(2.46–9.65)
	1.28	10	8.11	5.49	10.46	0.84	36.74	(3.59–6.03)
	28.28	2	7.79	7.79	6.16	3.43	12.14	(5.61–9.96)
Cmax.irinotecan (ng/ml)	Total	27	4539.57	3560.00	5151.12	417.28	28,571.00	(2176.5–5537.58)
	1.1	15	5799.04	3992.00	6470.94	1690.00	28,571.00	(3224–5721.58)
	1.28	10	3291.13	2176.50	2165.35	417.28	6440.00	(2013–5282.5)
	28.28	2	1335.78	1335.78	1247.65	453.56	2218.00	(894.67–1776.89)
Cmax.SN38 (ng/ml)	Total	27	87.69	52.30	113.72	14.00	568.37	(36–79)
	1.1	15	48.95	37.70	35.28	14.00	140.19	(23–61.13)
	1.28	10	128.30	60.95	161.87	37.00	568.37	(47.14–126.25)
	28.28	2	175.23	175.23	169.32	55.50	294.96	(115.36–235.09)
Cmax.SN38G (ng/ml)	Total	27	202.10	190.51	127.65	54.50	628.00	(102.95–264)
	1.1	15	211.78	189.00	151.67	54.50	628.00	(102.9–278.17)
	1.28	10	198.36	215.50	100.38	71.60	383.34	(111.65–256.75)
	28.28	2	148.20	148.20	59.83	105.90	190.51	(127.05–169.36)

AUC values for irinotecan among the *1/*1 and *1/*28 patients were statistically different (Wilcoxon–Mann–Whitney test, *p* = 0.001) with lower values for the *1/*28 patients, who were given a lower dose (370 mg/m^2^ *vs.* 310 mg/m^2^) than the *1/*1 patients. Although higher doses meant greater exposure, no statistically significant difference was found for its active metabolite SN38 and its inactive metabolite SN38-G across the different populations (*1/*1, *1/*28, *28/*28).

### Efficacy outcomes

The patients were followed up until December 09, 2019. Median follow-up is 47 months (reverse Kaplan–Meier method, IC95% (38-NA)). In the entire population, progression occurred for 27 patients and median PFS was 14 months (range 1 to 65 months). Median PFS was 13 months (range 1 to 56 months) for *1/*1, 15 months (range 3 to 65 months) for *1/*28, and 35 months (range 10 to 35 months) for *28/*28.

Concerning OS, 22 deaths were observed in the entire population and median OS was 31 months (range 3 to 88 months). In the subgroups, there were 12 deaths in the *1/*1 group and median OS was 36 months (range 4 to 63 months), 10 deaths in the *1/*28 group and median OS was 27 months (range 3 to 88 months). Median OS was not reached in the 28*/28* group.

Among the 34 patients included, 14 (41%) achieved a partial response and 1 (3%) a complete response, giving an ORR of 44%. More precisely, we observed eight responders in the *1/*1 cohort, one of whom had a complete response (40%), five responders in the *1/*28 cohort (42%) and two responders in the *28/*28 cohort (100%). Nine patients were stable (26.5%): four in the *1/*1 cohort and five in the *1/*28 cohort. Progression occurred for seven patients (20.5%): six in the *1/*1 cohort and one in the *1/*28 cohort. Three were not assessable (8.8%). Among the patients with hepatic metastasis (*n* = 27), six underwent resection, five of whom were in the 1*/1* group and one in the 1*/28* group. The median response duration was 9 months in the 1*/1* group (range 0 to 22 months), 5 months in the 1*/28* group (range to 0 to 11 months), and 28 months the 28*/28* group (range 5 to 28 months).

## Discussion

A large number of studies have shown that some mCRC patients are more or less sensitive to the toxic effects of chemotherapy depending on their UGT1A1 polymorphism [6, 12–14]. Thus, irinotecan doses could be administered according to this genetic parameter rather than according to body surface area. In this context, our phase-II study aimed to evaluate the feasibility of irinotecan dose adjustment according to UGT1A1 polymorphisms for mCRC patients, in terms of toxicities and response.

In our population, the wild-type allele in UGT1A1 genes was the most common with 58.8% of mCRC patients, followed by heterozygous mutations, which were observed in 35.3%, and finally homozygous mutations, appearing in 5.9%. These data are consistent with that reported in the literature, with a prevalence of the wild-type allele (1*/1*) and a lesser frequency for the homozygous type (28*/28*) [6, 15].

It has been showed that OS is longer when RDI levels are at least 80% for patients with solid tumours treated with FOLFIRI [16]. In our population, 65% of the patients in the cohort *1/*1 and 42% of the patients in the cohort *1/*28 had an RDI of at least 85%. OS of *1/*1 cohort is higher than those of *1/*28 cohort, 36 and 27 months, respectively. So, our results seem to be consistent with this meta-analyses however our study lacks the power to confirm this. The adverse events appeared relatively similar between subgroups formed according to the irinotecan dose. Grade 3–4 neutropenia or febrile neutropenia occurred in three patients from the 1*/1* group (15%) and the 1*/28* (25%) group. This is consistent with previously published data suggesting that higher doses of irinotecan do not lead to higher rates of toxicity. Indeed, the results reported by Chen and colleagues (for Irinotecan 180 mg/m^2^) showed that grades 3–4 neutropenia occurred for 15% of the patients with wild-type UGT1A1 (1*/1*) and 30.8% of the patient with heterozygous UGT1A1 genotype (1*/28*) [17]. In Tsai’s study, irinotecan-related grades 3–4 adverse events were comparable between the control (no UGT1A1 genotyping, 180 mg/m^2^) and study groups (UGT1A1 genotyping before treatment, irinotecan dose escalation) [18]. Pàez et al. also concluded that there were no differences in grades 3–4 toxicities between the control group (180 mg/m^2^) and the high-dose group (300 mg/m^2^ for 1*/1* and 260 mg/m^2^ for 1*/28*) [19]. These data support the notion that high doses of irinotecan could be tolerated by patients with UGT1A1 wild-type or heterozygous variant (*1/*28). On the other hand, a recent single-case study conducted by Tsai and colleagues showed that homozygous patients (*28/*28) can received a fewer dose (i.e., 120 mg/m^2^) with both favourable clinical outcomes and toxicity profiles (*N* = 7) [20]. This will be interesting to investigate these results in a comparative case study with a population of patients with the same genetic characteristics.

The results of the pharmacokinetic analyses conducted in our study are similar to those of a research team from the Netherlands [21]. Indeed, they showed that patients receiving a reduced dose of irinotecan, based on UGT1A1 polymorphism, compared to the standard dose (i.e., 126 mg/m^2^ *vs.* 180 mg/m^2^) had slightly higher exposure to SN38, but that the incidence of toxicities (grade 4 neutropenia, febrile neutropenia, and diarrhoea) was comparable across the groups. In our study, we do not observed any significant difference in SN38 and SN38-G plasma exposure between patients treated with different doses of irinotecan adjusted according to their UGT1A1 polymorphism probably because of the small number of patients. However, the results suggest that patients treated with FOLFIRI could tolerate higher doses of irinotecan than the standard dose at 180 mg/m^2^ on the basis of UGT1A1 genotyping, with similar efficacy and without increased toxicities.

Our results concerning ORR are comparable to those reported in Toffoli’s phase-I study with the same dose of irinotecan based on UGT1A1 genotype, i.e., 43% of ORR (19 partial or complete responses among 44 assessable patients) [3].

Pàez and colleagues conducted a phase-II randomized study on 82 mCRC patients to evaluate the efficacy and safety of the high-dose irinotecan FOLFIRI regimen [19]. Patients with genotype 28*/28* were excluded. The control group received 180 mg/m^2^ of irinotecan. In the experimental group, 1*/28*patients and 1*/1* patients received 260 mg/m^2^ and 300 mg/m^2^, respectively. In the high-dose irinotecan group, the overall response rate was 67.5% against 43.6% in the control group (*p* = 0.001, OR 1.73, CI95 [1.03–2.93]). These rates were higher than those found in our population, i.e., 40.6% (for 1*/1* and 1*/28*). However, the authors pointed out that when BRAF-mutated tumours were considered, the ORR was 41.7% for the high-dose FOLFIRI regimen. We did not have information regarding BRAF mutations in our population to compare our results and to potentially explain our lower ORR.

Tsai and colleagues’ randomized controlled study showed that patients treated with a higher dose of irinotecan (biweekly) plus bevacizumab, had significantly better OS than those treated with 180 mg/m^2^ (30 months *vs.* 22 months, HR 0.693; 95% CI, 0.503 to 0.955; *p* = 0.02). In the control group, UGT1A1 genotyping was not conducted. In the study group, genotyping was performed before treatment initiation and patients received different doses depending on their genotype (180 mg/m^2^ for 1*/1* and 1*/28* and 120 mg/m^2^ for 28*/28*).Irinotecan dose escalation for this group was then based on AEs observed after two cycles and stopped if grade 3 or more AEs occurred. The maximum escalation was 260 mg/m^2^ for 1*/1*, 240 mg/m^2^ for 1*/28*, and 180 mg/m^2^ for 28*/28*. In our study, patients received a higher irinotecan dosage than in the study group formed by Tsai et al., i.e., 310 and 370 mg/m^2^, and median OS was 27 and 36, respectively. Although in our population, not all patients received targeted therapy in association with FOLFIRI, we found similar results for PFS: for the Tsai et al. study group, PFS was 14 months, and in our groups receiving higher doses of irinotecan, i.e., 310 and 370 mg/m^2^, it was 15 and 13 months, respectively.

A recent literature review of 14 studies concluded that mCRC with UGT1A1 wild-type or heterozygous variant can tolerate significantly higher irinotecan doses than conventional dose (310 to 390 mg/m^2^ for *1/*1; 260 to 350 mg/m^2^ for *1/*28). It increases the therapeutic effect without exacerbating toxicity [22].

In mCRC patients treated with FOLFIRI, it has been demonstrated that RDI has an impact on outcomes. Indeed, patients receiving at least ≥ 80% of RDI have significantly better PFS and OS than those receiving less than 80%. These results reinforce the importance of dosing adaptation according to UGT1A1 polymorphism both to prevent the toxicities that can lead to dose reduction or treatment delay and to maintain the highest RDI for the patient to obtain good survival.

One limitation to our study was the small population analysed. Because the final statistical analysis was conducted on n = 34 instead of the planned n = 47 patients required for the Bryant and Day design, the analysis was underpowered. This was partly because UGT1A1 genotyping is not carried out as routine practice but was mandatory for our study. The genotyping was conducted by an external laboratory and delivery of the results was sometimes long, so patients received a first cycle with the standard dose of irinotecan instead of the dose based on their genotype.

To conclude, our results provide additional arguments in favour of the feasibility of adaptation of the FOLFIRI regimen according to UGT1A1 genotype for mCRC patients. This strategy provides an optimisation of irinotecan dosage for *1/*1 and *1/*28 without increasing toxicity. Prospective randomized studies are warranted to assess the efficacy among patients liable to receive higher irinotecan doses than the standard dose on the basis of their UGT1A1 genotype.

## Data Availability

The datasets used and/or analysed in the current study could be made available from the corresponding author on reasonable request.
